# Zirconium Oxycarbide: A Highly Stable Catalyst Material for Electrochemical Energy Conversion

**DOI:** 10.1002/cphc.201900539

**Published:** 2019-07-10

**Authors:** Niusha Shakibi Nia, Daniel Hauser, Lukas Schlicker, Albert Gili, Andrew Doran, Aleksander Gurlo, Simon Penner, Julia Kunze‐Liebhäuser

**Affiliations:** ^1^ Leopold-Franzens-Universität Innsbruck Innrain 52c (Josef-Möller-Haus) A-6020 Innsbruck Austria; ^2^ Fachgebiet Keramische Werkstoffe/Chair of Advanced Ceramic Materials, Institut für Werkstoffwissenschaften und -technologien Technische Universität Berlin Hardenbergstr. 40 10623 Berlin Germany; ^3^ Advanced Light Source Lawrence National Laboratory Berkeley California 94720 USA

**Keywords:** electrocatalysis, hydrogen evolution reaction, online DEMS, transition metal carbides, zirconium oxycarbide

## Abstract

Metal carbides and oxycarbides have recently gained considerable interest due to their (electro)catalytic properties that differ from those of transition metals and that have potential to outperform them as well. The stability of zirconium oxycarbide nanopowders (ZrO_0.31_C_0.69_), synthesized via a hybrid solid‐liquid route, is investigated in different gas atmospheres from room temperature to 800 °C by using in‐situ X‐ray diffraction and in‐situ electrical impedance spectroscopy. To feature the properties of a structurally stable Zr oxycarbide with high oxygen content, a stoichiometry of ZrO_0.31_C_0.69_ has been selected. ZrO_0.31_C_0.69_ is stable in reducing gases with only minor amounts of tetragonal ZrO_2_ being formed at high temperatures, whereas it decomposes in CO_2_ and O_2_ gas atmosphere. From online differential electrochemical mass spectrometry measurements, the hydrogen evolution reaction (HER) onset potential is determined at −0.4 V_RHE_. CO_2_ formation is detected at potentials as positive as 1.9 V_RHE_ as ZrO_0.31_C_0.69_ decomposition product, and oxygen is anodically formed at 2.5 V_RHE_, which shows the high electrochemical stability of this material in acidic electrolyte. This peopwery makes the material suited for electrocatalytic reactions at anodic potentials, such as CO and alcohol oxidation reactions, in general.

## Graphical abstract

**Stable nanopowders**: The electrochemical behavior of ZrO_0.31_C_0.69_ is studied in acidic electrolyte over a wide potential range. The onset potential of the hydrogen evolution reaction is −0.4 V_RHE_. CO_2_ formation due to anodic decomposition of ZrO_0.31_C_0.69_ is detected at 1.9 V_RHE_, accounting for the oxycarbide's high electrochemical stability. Thus, the oxycarbide is generally suited for anodic electrocatalytic reactions, such as CO and alcohol oxidation.

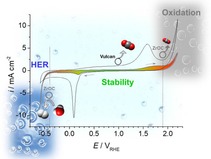

## 1 Introduction

The interstitial alloys of transition metals, known as metal carbides, nitrides and oxycarbides, have been subject to numerous studies because of their promising chemical and physical properties which differ from those of their parent metals.^1^, ^2^, ^3^ It has been reported that the interstitial incorporation of carbon atoms in the lattice of early transition metals from groups 4–6 result in the modification of their structural^2^, ^4^ and electronic properties, and their chemical activity,^2^, ^3^, ^6^ which mainly leads to reactivities similar to Pt‐group metals.^5^ Transition metal carbides (TMCs) are known to be suitable catalysts for reforming processes such as the hydrogenation of carbon monoxide and the water gas shift (WGS) reaction.^7^ The promising electrochemical stability of early TMCs over wide potential and pH ranges confirms their potential for use as low cost electrocatalysts in several electrochemical reactions.^8^, ^9^ Accordingly, they have shown interesting electrocatalytic performance in the oxidation of C1 (e. g. CO, CH_3_OH) and C2 (e. g. CH_3_CH_2_OH) molecules,^10^ as well as in reduction reactions, such as the hydrogen evolution reaction (HER)^6^, ^11^, ^12^, ^13^, ^14^ and the oxygen reduction reaction (ORR).^15^ More specifically, an improved electrocatalytic activity towards the HER was demonstrated for tungsten carbides (WC, W_2_C)^11^, ^12^ in comparison to molybdenum carbide (Mo_2_C)^12^, ^13^ and titanium carbide (TiC).^14^ In some cases, TMCs are even counted among promising alternatives to noble‐metal catalysts.^16^ For example, Mo_2_C has been reported to show a better activity than noble‐metals towards the selective gas‐phase conversion of CO_2_ to CO by H_2_.^17^ Due to their stability under reductive conditions, TMCs have been discussed as potential electrode materials for the electrochemical reduction of carbon dioxide as well.^18^

Composites of metal carbides with noble metals have also high potential for application in catalysis and electrocatalysis, as has been shown in numerous examples.^6^ TMC surface modification through the deposition of a monolayer of Pt can for instance enhance the HER activity of WC,^19^ TiC,^14^ and niobium carbide (NbC).^20^

Most of the TMC materials have the intrinsic tendency to form oxycarbides due to their high affinity to oxygen.^21^, ^22^, ^23^ Among oxygen modified TMCs, the synthesis and characterization of titanium oxycarbide (TiO_x_C_y_) powders and flat films as innovative catalyst support materials to replace carbon have been thoroughly studied over the last decade.^24^ TiO_x_C_y_ supported Pt nanoparticles showed enhanced catalytic activity towards the oxidation of ethanol, suggesting this material as potential alternative to carbon for use as support in direct ethanol fuel cells (DEFCs).^25^ Recently, differential electrochemical mass spectrometry (DEMS) investigations of the ethanol oxidation reaction (EOR) in acidic electrolytes have shown that TiO_x_C_y_ is a synergistic support for Pt nanoparticles and significantly enhances the CO_2_ conversion efficiency at room temperature.^26^ As another example, the synthesis and characterization of zirconium oxycarbide powders have been reported in various studies^27^, ^28^, ^29^, ^30^. In a recent work, phase pure zirconium oxycarbide nanopowders (ZrO_x_C_y_) of defined C/Zr ratio have been successfully synthesized via a hybrid solid‐liquid synthesis route,^30^ which resulted in an electrode material with good electrical conductivity and thus makes ZrO_x_C_y_ nanopowders an interesting material for electrocatalysis studies as well.

Due to the fact that TMCs are a very promising materials class for use in electrocatalysis and because of their tendency to form oxycarbides under specific working conditions,^21^, ^22^, ^23^ it is of high interest to determine the intrinsic electrocatalytic activity of oxycarbide materials for some of the most important electrocatalytic reactions. In this respect, it is highly important to also screen the stability of the (oxy)carbides under the electrochemical working conditions of interest. At the same time, their stability under specific synthesis conditions in the gas phase is essential, because it determines the overall materials properties, such as its surface chemistry, morphology, structure, and electronic structure, which will in turn have a great influence on the (electro)catalytic properties.

In the present paper, the stability of ZrO_x_C_y_ nanopowders^30^ with a stoichiometry of ZrO_0.31_C_0.69_ is studied in the gas phase by using in‐situ X‐ray diffraction (XRD) and in‐situ electrical impedance spectroscopy (EIS). The ZrO_0.31_C_0.69_ powder is then electrochemically investigated in 0.5 M H_2_SO_4_ in terms of its stability in acidic electrolyte and its electrocatalytic activity. Online DEMS studies are performed to determine the material's electrocatalytic activity towards the HER and to evaluate its electrochemical stability in a wide anodic potential range. All electrochemical potentials are given versus the reversible hydrogen electrode (RHE).

## 2 Results and Discussion

**Figure 1:**
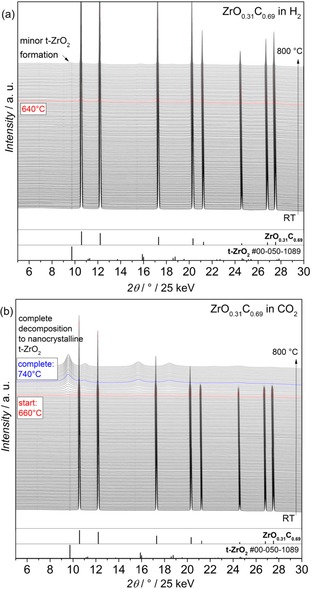
In‐situ collected X‐ray diffractograms of ZrO_0.31_C_0.69_ in different gas atmospheres: a) pure H_2_ and b) pure CO_2_. Temperature range: from room temperature (25 °C) to 800 °C, heating ramp: 10 °C min^−1^ and gas flow: 10 mL min^−1^.

The bulk structural stability of ZrO_0.31_C_0.69_ powder has been analyzed by synchrotron‐based in‐situ X‐ray diffraction experiments at beamline 12.2.2 of the Advanced Light Source, Berkeley, California,^31^, ^32^ in different reductive and oxidative gas environments. The powder is treated under flowing conditions at a flow of 10 mL min^−1^. Figure 1 depicts the respective diffractograms collected at ambient H_2_ and CO_2_ pressures that allow to follow possible phase transitions of ZrO_0.31_C_0.69_ to ZrO_2_ and ZrC. Figure S1 (in the Supporting Information) shows diffractograms of ZrO_0.31_C_0.69_ in Ar/H_2_, He, O_2_ and CH_4_ (ambient pressure). Generally, ZrO_0.31_C_0.69_ is stable under reductive reaction conditions, as in pure H_2_ (Figure 1a) or in Ar diluted H_2_ (Figure S1a), because only small amounts or no tetragonal ZrO_2_ (t‐ZrO_2_) form. Under quasi‐inert reaction conditions, such as in He gas, also only minor amounts of tetragonal ZrO_2_ are formed at high temperatures (see Figure S1b). The formation onset for ZrO_2_ formation in these minor amounts can be determined at 640 °C in pure H_2_ and at 730 °C in pure He. No tetragonal ZrO_2_ has been observed in Ar diluted H_2_. A comparable decomposition temperature of 780 °C is found in pure CH_4_ (Figure S1d). A different behavior is observed for ZrO_0.31_C_0.69_ in CO_2_ (see Figure 1b), where complete decomposition into ZrO_2_ is observed. The decomposition process takes place over a rather large temperature region of 70–100 °C, starting at 660 °C (being complete at 740 °C), where tetragonal ZrO_2_ is formed. Details of this process are explained in the supporting information.

**Figure 2:**
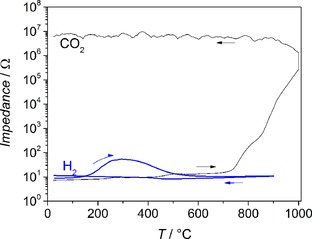
In‐situ electrical impedance spectroscopy measurements on ZrO_0.31_C_0.69_ in CO_2_ (black line) and in H_2_ (blue line).

To confirm the in‐situ XRD results and specifically the formation of ZrO_2_, additional in‐situ EIS investigations have been carried out. It is expected that the electric conductivity of the oxycarbide material changes as a function of gas atmosphere and a semimetal‐semiconductor transition potentially takes place upon the partial transformation to ZrO_2_.^33^ According to Figure 2 and Figure S2, exactly this behavior has been observed. In H_2_, a slight increase in impedance is observed at around 170–500 °C, most likely due to the formation of minor amounts of tetragonal ZrO_2_. It is expected that ZrO_2_ formation starts at the materials surface,^21^ which is first affecting the impedance of the surface and occurs at a higher temperature in the bulk material, as probed with XRD. Interestingly, the impedance decreases again at temperatures >500 °C in H_2_, which might be due to an increase of ionic conductivity at higher temperatures that can only be reached if just minor amounts of ZrO_2_ are formed. In He, the metallic conductivity (with values between 7–50 Ω) is preserved during the temperature cycle from room temperature to 800 °C (Figure S2).

In contrast, during annealing in CO_2_, a semimetal to semiconductor/insulator transition is observed, where the impedance rises by several orders of magnitude to about 10^7^ Ω and stays at this high value after the first increase (see Figure 2). In good agreement with the in‐situ XRD results, the onset of the major impedance increase in CO_2_ is determined at 700 °C, while a small impedance increase already sets in at temperatures >400 °C.

**Figure 3:**
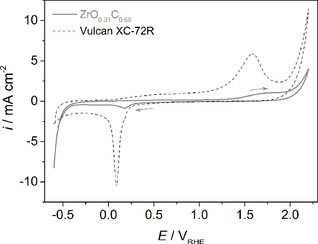
CVs of ZrO_0.31_C_0.69_ and carbon Vulcan XC‐72R recorded in 0.5 M H_2_SO_4_ at room temperature. Scan rate: 10 mV s^−1^. Current densities are given with respect to the geometric surface areas.

To investigate the electrochemical behavior of ZrO_0.31_C_0.69_, cyclic voltammograms (CVs) are recorded over a wide potential range from −0.6 V to 2.2 V, with an admission potential of 0 V (see Figure 3). In Figure 3, all faradaic current densities are given with respect to the geometrical surface area to be comparable to the relevant literature. At positive potentials, a small oxidation peak can be identified at ∼1.6 V followed by a steady increase in anodic current. In the negative going scan, a reduction peak is observed at 0.16 V; high cathodic currents are measured at potentials more negative than −0.4 V. Since the surface chemistry of ZrO_0.31_C_0.69_ is reported to be dominated by oxidized carbon and carbonate species in addition to zirconia,^30^ the electrochemical behavior of a carbon reference material (Vulcan XC‐72R) is also evaluated under thesame conditions (see Figure 3).

Generally, higher faradaic current densities are measured with carbon Vulcan XC‐72R, which is visible after normalization of the current with respect to the geometrical (see Figure 3) and the BET surface areas of Vulcan (250 m^2^ g^−1^)^34^ and ZrO_0.31_C_0.69_ (350 m^2^ g^−1^) ^[30]^ (see Figure S3, supporting information). The differences in the CVs show that the ZrO_0.31_C_0.69_ cannot be entirely covered with the excess carbon from the synthesis process.^30^ Comparison of the cathodic potential region from −0.6 V to 0 V for both ZrO_0.31_C_0.69_ and carbon shows that the ZrO_0.31_C_0.69_ is more active in the potential region where the HER takes place, resulting in an earlier HER onset potential and in higher current densities compared to carbon. At anodic potentials of >1.8 V carbon shows high anodic currents with an earlier onset compared to that of ZrO_0.31_C_0.69_, suggesting an active dissolution of carbon at these high potentials. Two distinct peaks can be identified from the CV of carbon, an oxidation peak at 1.58 V with its corresponding reduction peak at 0.09 V. The position of the oxidation peak determined from the CV of carbon being only 20 mV different from the one identified from the CV of ZrO_0.31_C_0.69_ suggests that the origin of this peak could be related to the presence of carbon species in the ZrO_0.31_C_0.69_ material.

**Figure 4:**
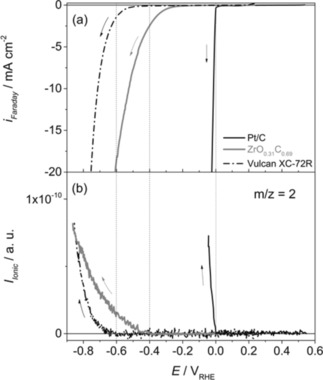
a) LSVs of Pt/C, ZrO_0.31_C_0.69_, and Vulcan XC‐72R recorded during the HER in 0.5 M H_2_SO_4_ at room temperature. b) MSLSVs for m/z=2. Scan rate: 5 mV s^−1^. Dotted lines: HER onset potentials. Current densities are given with respect to the geometric surface areas.

For a better comparison of the electrocatalytic activity of both materials towards the HER, linear sweep voltammetry (LSV) measurements were performed in a potential range from 0.2 V to −0.86 V, and the corresponding mass spectrometric linear sweep voltammograms (MSLSVs) were recorded at m/z=2 to determine the onset potential of hydrogen evolution at both materials (see Figure 4). Additionally, reference measurements were carried out using Pt/C (20 wt% of Pt) catalyst powder synthesized by carbonyl chemical route (CCR),^26^ where the potential was swept from 0.5 V to −0.06 V.

The onset potential of the HER is identified by the increase of the ionic current at m/z=2 and a steep increase in the cathodic faradaic current density. The onset of the HER for Pt/C is located at 0.00 V versus RHE, as expected. In case of ZrO_0.31_C_0.69_ and carbon Vulcan the cathodic current increase takes place at more cathodic potentials compared to Pt/C. An accurate analysis of the HER onset potential from the ionic currents confirms that the increase in m/z=2 takes place at −0.4 V and −0.6 V at ZrO_0.31_C_0.69_ and carbon Vulcan, respectively (see Figure 4b). Since the faradaic currents at ZrO_0.31_C_0.69_ and carbon Vulcan incline at less negative potentials than the HER onset potential determined from DEMS, the contribution of surface reactions to the observed faradaic currents cannot be excluded. This means that the significant faradaic currents measured at low overpotentials of <−0.4 V at ZrO_0.31_C_0.69_ and Vulcan are not related to the HER. Interestingly, the determined overpotential of the HER on ZrO_0.31_C_0.69_ (−0.4 V) approaches −0.6 V at a current density of 20 mA cm^−2^. In agreement with our previous assumption, this deviation could be rationalized through the contribution of surface reactions taking place simultaneously to the HER, which is currently being investigated and will be reported separately.

**Figure 5:**
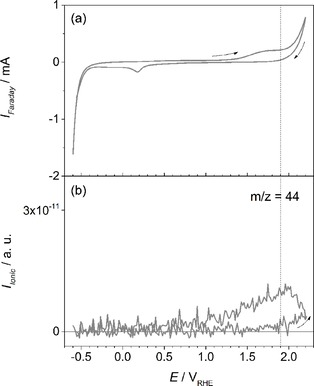
a) CV of ZrO_0.31_C_0.69_ recorded in 0.5 M H_2_SO_4_ at room temperature. b) MSCV for m/z=44. Scan rate: 10 mV s^−1^.

It has been found that important decomposition products of oxycarbide materials caused by anodic oxidation are CO_2_ and CO.^10^, ^22^ To evaluate the electrochemical stability of ZrO_0.31_C_0.69_, mass spectrometric cyclic voltammograms (MSCVs) were recorded at m/z=44 to determine the onset potential for carbon dioxide (CO_2_) formation in a potential range from −0.6 V to 2.20 V (Figure 5). The onset potential for CO_2_ formation can be determined as ∼1.9 V where the ionic current at m/z=44 deviates from the background signal (Figure 5b). This signal continues to increase in the backward scan and peaks at 2.0 V, before it decreases again. The absence of CO_2_ formation between 1.2 V and 1.7 V, where an increase in the faradaic current is visible (Figure 5a), confirms that the anodic currents in this potential region should be related to other surface reactions than oxidation of ZrO_0.31_C_0.69_ to CO_2_.

Additional measurements under the same conditions were performed with carbon Vulcan (see Figure S4), according to which an early onset potential of nearly 1.0 V is determined for the evolution of CO_2_ that is perfectly in line with the faradaic current increase at the same potential. This confirms that the anodic peak at 1.6 V can be solely related to CO_2_ formation due to the oxidation of carbon Vulcan.

**Figure 6:**
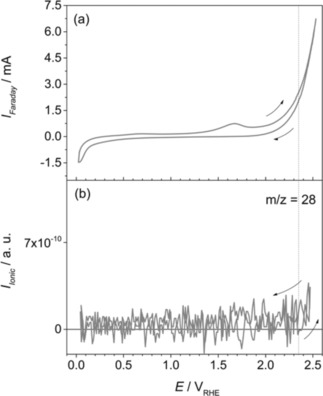
a) CV of ZrO_0.31_C_0.69_ recorded in 0.5 M H_2_SO_4_ at room temperature. b) MSCV for m/z=28. Scan rate: 20 mV s^−1^.

Further investigations of the electrochemical stability of ZrO_0.31_C_0.69_ were performed by increasing the anodic potential progressively to higher values than 2.2 V. Figure 6 depicts the CV recorded between 0.03 V and 2.5 V. It was observed that by applying potentials higher than 2.2 V, carbon monoxide (CO) formation can be detected from ionic currents at m/z=28 in addition to higher amounts of CO_2_ detected at m/z=44. The onset potential for CO formation was determined at 2.35 V.

At potentials >2.35 V, the ionic current signal at m/z=28 increases and reaches a maximum at 2.5 V in the forward scan and then decreases in the backward scan. It is noteworthy that possible contributions of the CO_2_ formation through its mass fragment at m/z=28 have been experimentally determined and subtracted from the MSCV depicted in Figure 6b. Since the signal at m/z=28 does not follow the features of m/z=44 (Figure 5b), contributions of the ion current signals from the same parent molecule can be excluded. CO formation at E>2.2 V can be related to further degradation of the oxycarbide material at these high anodic potentials in acidic electrolyte.

**Figure 7:**
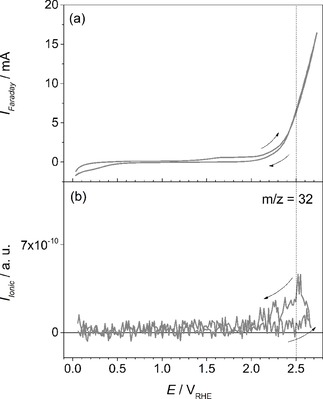
a) CV of ZrO_0.31_C_0.69_ recorded in 0.5 M H_2_SO_4_ at room temperature. (b) MSCV for m/z=32. Scan rate: 20 mV s^−1^.

Further increase of the anodic potential from 2.5 V to 2.7 V is depicted in Figure 7. In addition to CO_2_ and CO detection, oxygen evolution has been detected at these high anodic potentials via ionic currents at m/z=32. In Figure 7b, one can see that O_2_ is continuously formed between 2.5 V and 2.7 V in the forward and backward scan. A decrease in the ionic signal is visible at E<2.5 V in the backward scan.

## 3 Conclusions

The gas phase stability of zirconium oxycarbide nanopowders with a stoichiometry of ZrO_0.31_C_0.69_ is studied in different gas atmospheres. In‐situ XR diffractograms collected from room temperature to 800 °C show that the oxycarbide material is stable under reductive conditions in H_2_ and in Ar diluted H_2_, as well as in He and in CH_4_, with only small amounts of tetragonal ZrO_2_ formed at high temperatures, while it is oxidized in O_2_ and CO_2_ with a complete decomposition of ZrO_0.31_C_0.69_ to ZrO_2_. The metallic conductivity of ZrO_0.31_C_0.69_ is preserved if minor amounts of ZrO_2_ are formed; in case of complete decomposition, a severe increase of impedance is observed. The overpotential of the electrochemical HER at ZrO_0.31_C_0.69_ is determined at −0.4 V from DEMS analysis, which is a reasonable value compared to other TMC catalyst materials for this reaction, such as Mo_2_C (−0.09 V) ^[13]^ and WC (−0.184 V).^12^ It is noteworthy that the HER onset potential determination is not always performed by tracking the actual H_2_ gas evolution onset with DEMS (as in our case, and e. g. in^13^), but through evaluation of the faradaic current responses only. This may result in inaccuracies associated with cathodic currents that are not related to the hydrogen evolution, and that are often observed for carbide and oxycarbide electrodes. It is assumed that surface composition changes during the HER contribute to the significant faradaic currents measured at low overpotentials of < −0.4 V at this material, which is an effect that will be further investigated in the future. The decomposition of ZrO_0.31_C_0.69_ to CO_2_ and CO is detected at very positive potentials of 1.90 V and 2.35 V, respectively, which shows the material's high stability in the anodic potential region. Therefore, ZrO_0.31_C_0.69_ is generally well suited as catalyst or catalyst support for electrochemical energy conversion reactions at anodic potentials, such as alcohol and CO oxidation.

## Experimental Section

The ZrO_0.31_C_0.69_ powder was synthesized via a gel – solid state hybrid synthesis routine. Details have been previously published.^30^ To feature the properties of a structurally stable Zr oxycarbide with high oxygen content, a stoichiometry of ZrO_0.31_C_0.69_ has been selected. Note that according to literature, only Zr (oxy)carbides in the ratio range between C/Zr=0.64 and C/Zr=0.98 are stable.^35^ BET experiments have been conducted on a Quantachrome Nova 2000 Surface Area and Pore Size Analyzer in a standard procedure by adsorption of liquid nitrogen at 77 K. According to BET/BJH measurements, the oxycarbide powder (including carbon excess) has no pores with a specific surface area of 350 m^2^ g^−1^.

A carbon (Vulcan XC‐72R, Cabot Corporation, Austria) support decorated with 20 wt% of Pt (Pt/C) via the carbonyl chemical route^26^ was used as reference for DEMS calibration. For the electrochemical measurements, a powder ink was prepared by mixing 2–3 mg of the powders with 15 μL of Nafion solution (5 wt%, Sigma Aldrich) and 0.5 mL of de‐ionized (DI) water (Merck, Germany, Milli‐Q 18.2 MΩ).

The in‐situ high temperature synchrotron XRD experiments in different gas atmospheres were performed at the beamline 12.2.2, Advanced Light Source (ALS), Lawrence Berkeley National Lab, California. The in‐situ diffraction patterns were collected in the angle‐dispersive transmission mode with a focused 25 keV monochromatic beam (λ=0.4959 Å, 30 μm spot size diameter). The ZrO_0.31_C_0.69_ sample powder was heated in a 0.7 mm quartz capillary under a continuous gas flow (10 mL min^−1^) injected through a 0.5 mm tungsten tube. The capillary is heated at 10 °C min^−1^ to 800 °C in an infrared‐heated SiC tube furnace, as described in References [31, 32]. Diffraction patterns were recorded by a Perkin Elmer flat panel detector (XRD 1621, dark image and strain correction) every 35 seconds during the heating cycle. The detector raw data was integrated with Dioptas software,^36^ the analysis of the diffractograms was carried out using reference data from the ICDD database.^37^

The in‐situ impedance cell consists of an outer quartz tube with two inner quartz tubes to which the sample and the electrodes are attached. Heating is provided by a tubular Linn furnace and controlled by a thermocouple (K‐element), located in the reactor about 5 mm downstream of the sample, and a Micromega PID temperature controller. The impedance is measured by an IM6e impedance spectrometer (Zahner Messsysteme), which provides data on the impedance and the phase angle of the current as a function of voltage. The ZrO_0.31_C_0.69_ sample was pressed into pellets with a pressure of 250 MPa (5 mm diameter, thickness ∼0.2 mm, sample mass about 20 mg) and placed between two circular Pt electrodes, effectively forming a plate capacitor in mechanically enforced contact with the sample pellet. For all temperature‐programmed impedance/conductivity measurements described in this article, an excitation voltage of 20 mV and a frequency of 1 Hz were applied to the Pt electrodes.

The electrochemical stability and the electrocatalytic activity of powder materials towards the HER were studied by performing DEMS measurements with a Hiden HPR‐40 mass spectrometer. An electron energy of 70 eV was applied for ionization, and the employed emission current was 500 μA. The secondary electron multiplier detector was operated at 850 V and an acceleration voltage of 3 V was set for detection of all species. A commercial single flow DEMS cell (Type A, Hiden Analytical, U.K.) was used in a thin layer electrolyte configuration coupled with an Autolab PGSTAT 302N operating with Nova 2.0 software. An amorphous activated carbon (YP‐50F, Kuraray) mixed with Polytetrafluoroethylene (PTFE)‐bound (60 % dispersion in water, Sigma‐Aldrich, Germany) was employed as the counter electrode (CE) and the quasi reference electrode (QRE).^38^ 20–40 μL of the ink deposited on a glassy carbon support (5 mm of diameter, geometric surface area=0.196 cm^2^) was used as the working electrode (WE). A PTFE membrane (Gore‐Tex, 75 mm thickness, 50 % porosity, 0.02 μm pore diameter) was used as interface between the electrolyte and the high vacuum in the mass spectrometer and was placed in front of the WE. A stainless‐steel frit was supporting the membrane from the back, at the entrance to the high vacuum. Cyclic voltammetry (CV) and linear sweep voltammetry (LSV) techniques were used to determine the electrochemical properties of ZrO_0.31_C_0.69_ in an Argon (Ar, >99.999 %, Messer, Austria) saturated solution of aqueous 0.5 M H_2_SO_4_ (96 %, Suprapur, Merck, Germany) with a pH of 0.25. The response time of the mass spectrometer was calibrated with the Pt/C catalyst powder (see Figure S5) in the same electrolyte.

## Conflict of interest

The authors declare no conflict of interest.

## Supporting information

As a service to our authors and readers, this journal provides supporting information supplied by the authors. Such materials are peer reviewed and may be re‐organized for online delivery, but are not copy‐edited or typeset. Technical support issues arising from supporting information (other than missing files) should be addressed to the authors.

Supplementary
