# Supplementary material for: Zirconium Oxycarbide: A Highly Stable Catalyst Material for Electrochemical Energy Conversion
Source: Chemphyschem. 2019 Jul 10;20(22):3067–73. doi: 10.1002/cphc.201900539 (PMC6900196; doi:10.1002/cphc.201900539)
Supplement: Supplementary file 1 — Supplementary [file CPHC-20-3067-s001.pdf]

CHEMPHYSCHEM

Supporting Information

© Copyright Wiley-VCH Verlag GmbH & Co. KGaA, 69451 Weinheim, 2019

Zirconium Oxycarbide: A Highly Stable Catalyst Material for Electrochemical Energy Conversion

Niusha Shakibi Nia, Daniel Hauser, Lukas Schlicker, Albert Gili, Andrew Doran, Aleksander Gurlo, Simon Penner,* and Julia Kunze-Liebhäuser*© 2019 The Authors. Published by Wiley-VCH Verlag GmbH & Co. KGaA.
This is an open access article under the terms of the Creative Commons Attribution License, which permits use, distribution and reproduction in any medium, provided the original work is properly cited.An invited contribution to a Special Issue on Electrocatalysis

Supporting Information

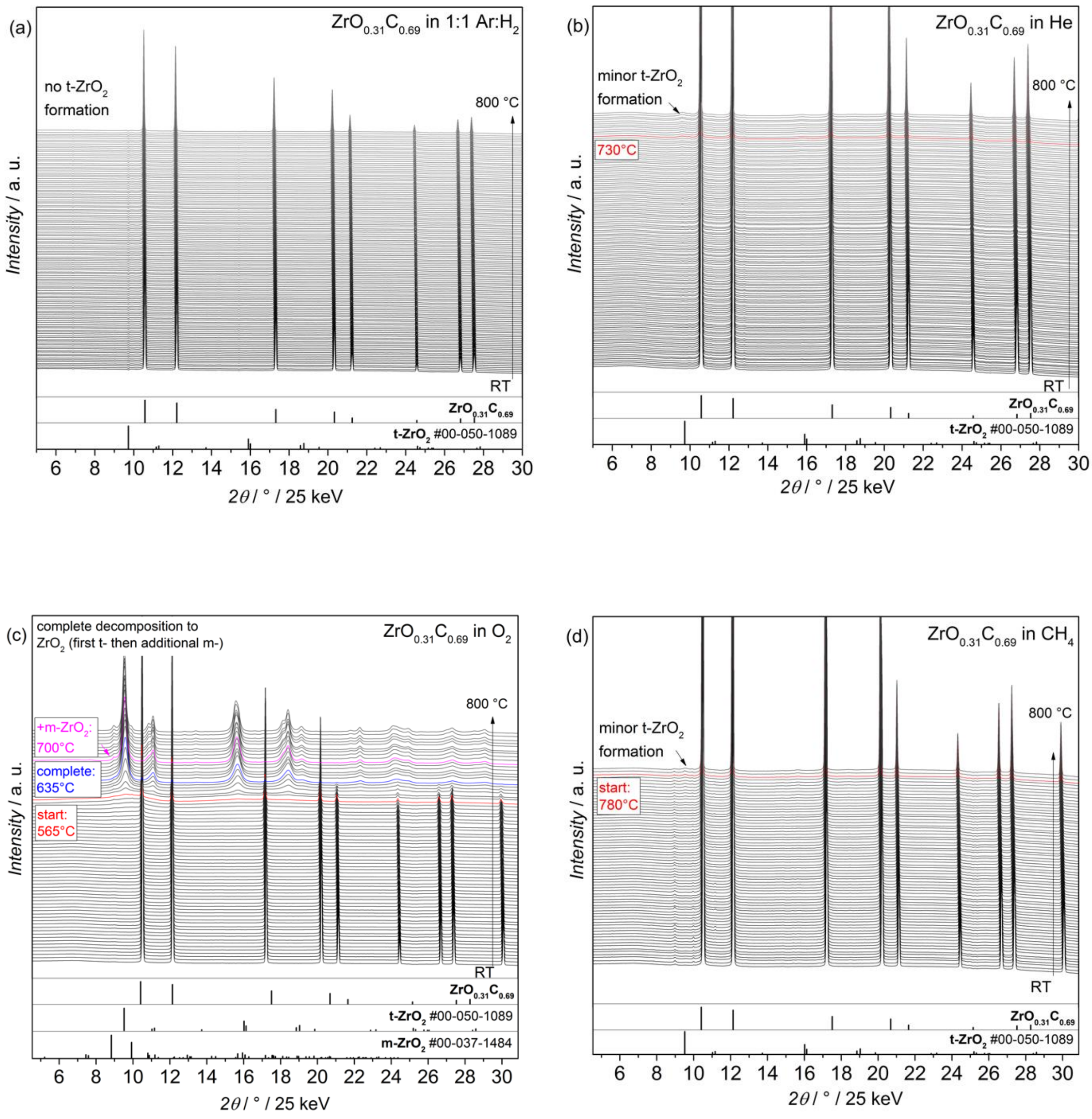

Figure S1: *In situ* X-ray diffractograms of $ZrO_{0.31}C_{0.69}$ in different gas atmospheres. (a) 1:1 Ar:H_2, (b) pure He, (c) pure O_2 and (d) pure CH_4. Temperature range: from room temperature (25 °C) to 800 °C, heating ramp: 10 °C min^{-1}.

The decomposition of $ZrO_{0.31}C_{0.69}$ in different gas atmospheres is strongly influenced by the polymorphism of ZrO_2, i.e. the existence of (meta)stable monoclinic (m-ZrO_2) and tetragonal ZrO_2 (t-ZrO_2). The transformation of the latter into the thermodynamically more stable monoclinic modification is itself a strong function of the gas atmosphere. It is known, that tetragonal ZrO_2 is stabilized either by structural defects or by grain size control. In that respect, the transformation is suppressed in strongly reducing atmospheres. The more oxidizing the atmosphere, the faster the transformation. With respect to our study, we note that in O_2, first t-ZrO_2 appears, then very fast also

m-ZrO_2 is observed. In CO_2, a similar process is found, but with delayed transformation into m-ZrO_2. Nevertheless, the temperature period between the first appearance of t-ZrO_2 and full decomposition is about the same as in O_2 (start at 565 °C, end at 635 °C, i.e. 70 °C) and in CO_2 (start at 660 °C, end at 740 °C, i.e. 80 °C)). The crystallization of t-ZrO_2 itself is a kinetically determined process, where nuclei of t-ZrO_2 have to be formed, which then facilitate the further crystallization.

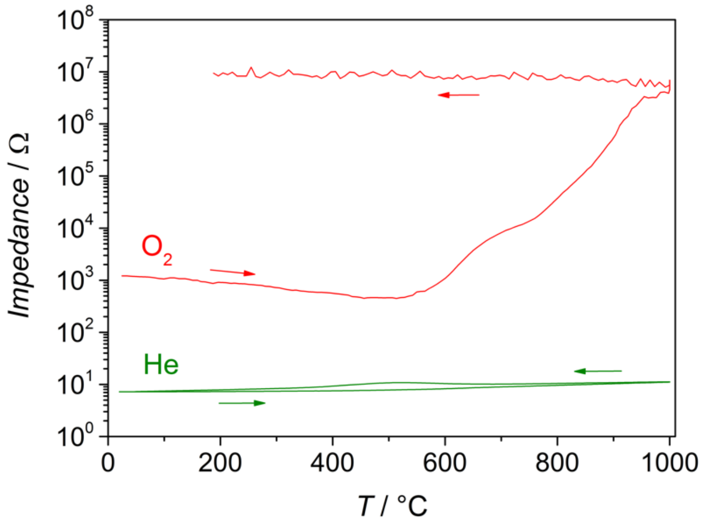

Figure S2: In situ electrical impedance spectroscopy measurements on $ZrO_{0.31}C_{0.69}$ in O_2 (red line) and in He (green line).

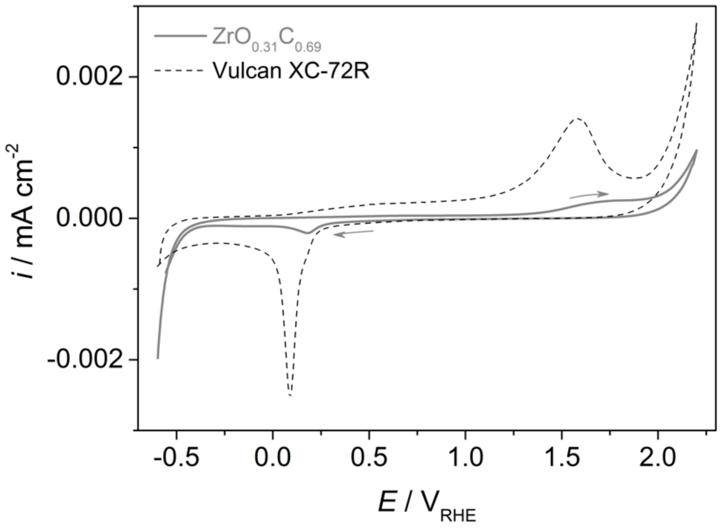

Figure S3. CVs of $ZrO_{0.31}C_{0.69}$ and carbon Vulcan XC-72R recorded in 0.5 M H_2SO_4 at room temperature. Scan rate: 10 mV s^{-1}. Current densities are given with respect to the BET surface areas.

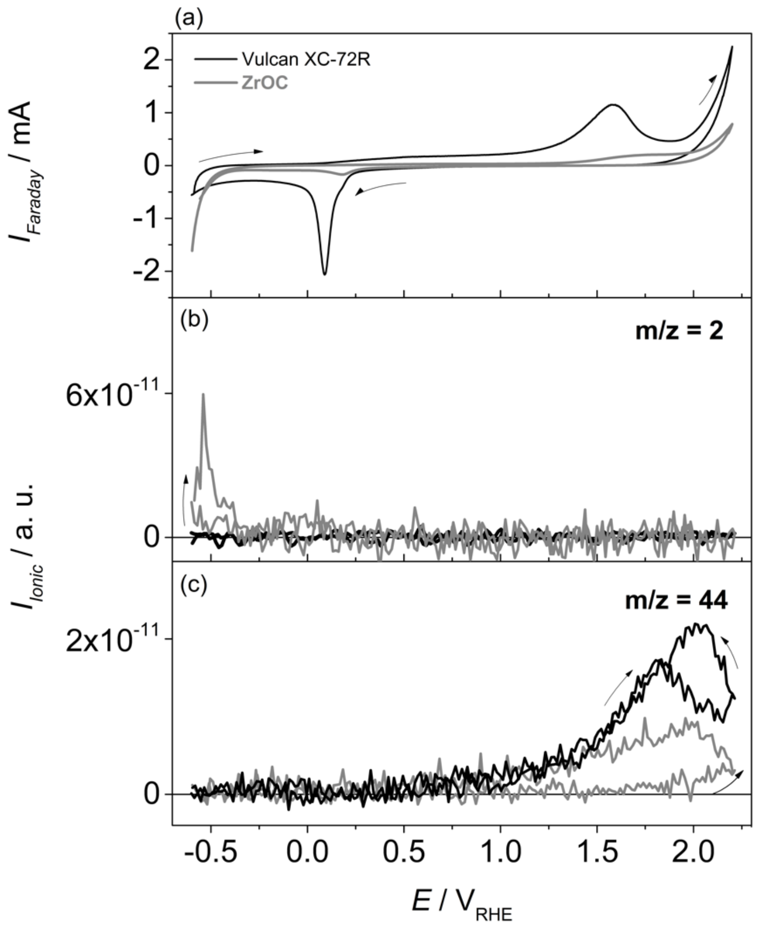

Figure S4: (a) CVs of $ZrO_{0.31}C_{0.69}$ and carbon Vulcan XC-72R recorded in 0.5 M H_2SO_4 at room temperature, (b) MSCVs for m/z = 2 and (c) MSCVs for m/z = 44. Scan rate: 10 mV s^{-1}.

DEMS calibration with Pt/C powder catalyst

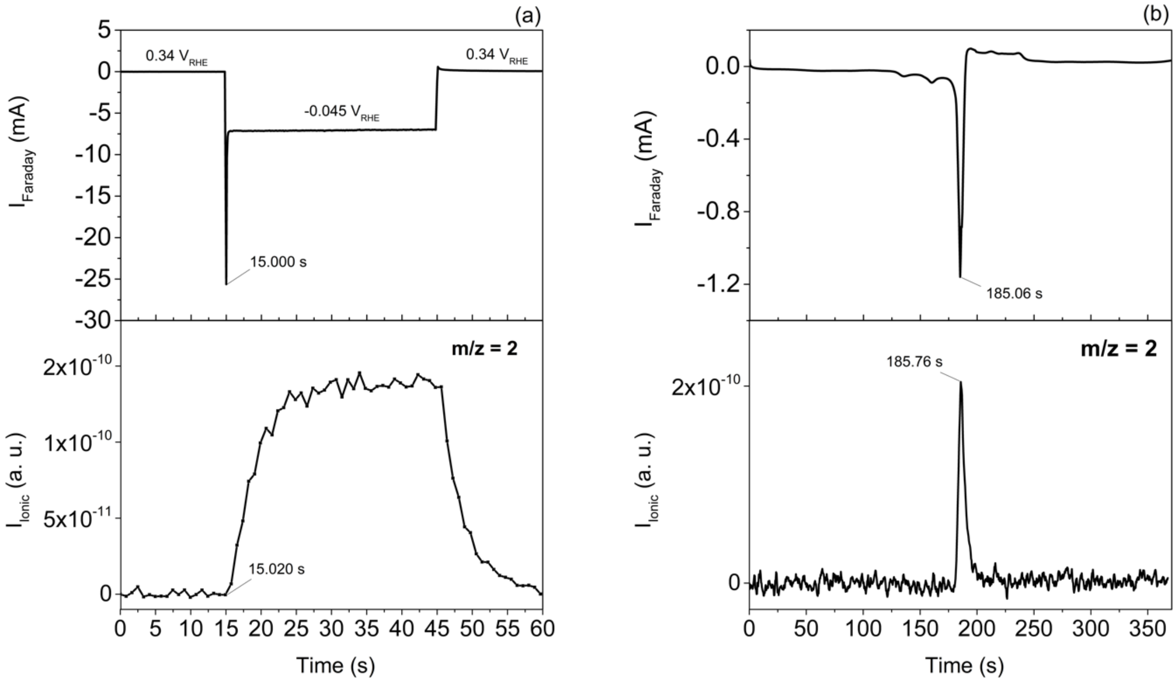

Figure S5: (a) Current transients and corresponding MSCTs at m/z = 2, (b) potentiodynamic measurements of current form 0.16 V to -0.02 V at 5 mV s^{-1} and corresponding mass spectrometric signal at m/z = 2 recorded with Pt/C catalyst in 0.5 M H_2SO_4 at room temperature.

The DEMS flow cell configuration was calibrated using Pt/C catalyst ink. To measure the current transients for hydrogen evolution at Pt/C (Figure S5a), the potential was stepped from 0.34 V to -0.045 V (vs RHE) while the mass spectrometric current transients (MSCTs) of the hydrogen signal (m/z = 2) were recorded online over time. With this technique, the delay time of hydrogen detection at m/z = 2 for potentiostatic experiments was determined as 0.02 s.

Additionally, the potential was screened between 0.16 V and -0.02 V with a scan rate of 5 mV s^{-1} (Figure S5b), from which a delay time of 0.7 s can be determined for potentiodynamic hydrogen detection. This delay time was used to correct the gas evolution onsets, determined under the same conditions, in the present manuscript.
